# Packaging of Cannabis Edibles, Health Warning Recall, and Perceptions Among Young Adults

**DOI:** 10.1001/jamanetworkopen.2025.3117

**Published:** 2025-04-03

**Authors:** Michael Cooper, Yuyan Shi

**Affiliations:** 1 Herbert Wertheim School of Public Health and Human Longevity Science, University of California, San Diego, La Jolla

## Abstract

**Importance:**

Cannabis edibles have increased in popularity, particularly among young adults and in states with recreational cannabis legalization. Little is known about the association of cannabis packaging regulations with health warning recall and perceptions.

**Objective:**

To evaluate whether features of packaging are associated with ability to recall health warnings as well as perceptions of cannabis edible products among young adults.

**Design, Setting, and Participants:**

This cross-sectional study was conducted from October 6 to 18, 2023, in 23 states and Washington, DC, that legalized recreational cannabis. Participants included young adults aged 18 to 29 years, including cannabis users and nonusers.

**Exposures:**

In the between-individual 3 (packaging styles) × 3 (health claims) × 7 (health warning themes) full-factorial randomized online experiment, participants viewed an image of the packaging of cannabis edible products with random variations in package styles (plain, normal branded, and youth-appealing packaging), health claims (none, pain relief, and sleep aid), and health warning themes (long-lasting effects, pregnancy-related harms, driving impairment, mental health problems, harms to youths, harms of high potency products, and delayed effects).

**Main Outcomes and Measures:**

Five self-reported outcomes, including warning recall (ability to correctly recall the warning label shown), product appeal rating, relative harm, adult-oriented appearance, and target age group.

**Results:**

Participants comprised 4500 young adults aged 18 to 29 years, including 3000 cannabis users and 1500 nonusers. These participants included 2287 men (50.8%); 1095 participants (24.3%) were aged 18 to 20 years, and 1905 (42.3%) were aged 21 to 25 years. Compared with normal branded packaging, plain packaging was associated with increased odds of correct warning recall (correct rate, 52.9%; odds ratio [OR], 1.47 [95% CI, 1.27-1.70]), decreased appeal ratings (mean appeal rating, 5.2 of 10; OR, 0.70 [95% CI, 0.61-0.80]), and increased perceived relative harm (mean harm rating, 2.6 of 5; OR, 1.48 [95% CI, 1.27-1.74]). Compared with normal branded packaging, youth-appealing packaging was associated with increased appeal ratings (OR, 1.40 [95% CI, 1.20-1.64]), particularly among younger adults aged 18 to 20 years. Compared with no health claims, the pain relief and sleep aid claims were associated with increased appeal ratings (pain relief: OR, 1.31 [95% CI, 1.17-1.46]; sleep aid: OR, 1.36 [95% CI, 1.24-1.50]), particularly among past-year nonusers.

**Conclusions and Relevance:**

Results of this cross-sectional study of the features of cannabis edible packaging suggested that requiring plain packaging, prohibiting youth-appealing features, and regulating unsubstantiated health claims may improve warning recall and accurate harm perceptions among young adults. Future observational studies evaluating the effects of these regulations are recommended.

## Key Points

**Question:**

How is the packaging of cannabis edibles associated with ability to recall health warnings as well as perceptions among young adults?

**Findings:**

This cross-sectional study on the features of cannabis packaging included 4500 young adults and found that plain packaging was significantly associated with increased odds of correct warning recall, decreased product appeal, and increased perceived harm and that youth-appealing packaging was associated with increased product appeal. Health claims were significantly associated with increased product appeal.

**Meaning:**

Policymakers may require plain packaging, prohibit youth-appealing features, and regulate unsubstantiated health claims to improve warning recall and perceptions of cannabis edible products among young adults.

## Teaser

This cross-sectional study evaluates whether the features of cannabis edible packaging are associated with health warning recall and safety perceptions of cannabis edible products among young adults.

## Introduction

Recreational cannabis legalization has rapidly expanded in the US to over 24 states and Washington, DC. Noncombustible methods of cannabis use, particularly consuming edible cannabis products, have gained popularity.^1^ Data in 2020 suggested that 60.8% of past-year adult cannabis users in states with legalized cannabis used edibles, with 19.5% of recreational users and 23.8% of medical users choosing them as their primary administration method.^1,2^ In 2022, cannabis edibles accounted for 12.1% of the cannabis market share in states with legalized cannabis.^3^

Cannabis use by young people poses additional health risks. Nearly 40% of young adults aged 18 to 29 years used cannabis in the past year, the highest prevalence among all age groups.^4^ Long-term, regular cannabis use among young people is associated with heightened risks of altered brain development, psychosocial impairment, and poor educational outcomes compared with older adults.^5,6^ The use of cannabis edibles may reduce certain risks associated with combustible products and products with high potency. However, cannabis edibles have unique health risks, such as delayed onset and extended duration of psychoactive effects. Evidence from poison center calls and hospital emergency department visits suggest that cannabis edibles were involved in most cases of acute cannabis intoxication.^7,8,9^

Cannabis policymaking may learn from the extensive literature on tobacco and food packaging, which found that regulating packaging features, such as package styles, health claims, and health warnings, has proven to be effective in modifying health outcomes. With respect to the effects of packaging styles, cigarette packaging styles were associated with smokers’ appeal ratings and perceptions and mediated the effects of health warnings.^10,11,12^ Plain packaging of tobacco products was associated with lower appeal ratings, lower intentions to use, and increased salience and recall of health warnings.^13,14^ Youth-appealing packaging in the food industry was associated with changes in food preferences and food intake.^15^ With respect to health claims, cigarette package descriptors, such as “light,” “mild,” and “low tar,” were associated with perceptions of reduced harm and being easier to quit.^16^ Although these descriptors have been banned in many countries, the tobacco industry has substituted terms such as *natural* or *smooth* and used claims about enhanced filters to achieve similar effects.^17^ In the food industry, nutritional claims on packaging about fat, sugar, and energy content were associated with increased perceptions of food healthfulness, purchase intentions, and consumption.^18^ With respect to the associations of health warnings, strengthened text-based warnings on tobacco products promoted health knowledge and accurate risk perceptions and was associated with reduced unhealthy use behaviors.^11^ Graphic warnings had even greater associations with these outcomes.^19^ Text-based and graphic health warnings on food products communicated health risks of high sugar, obesity, and diabetes and reduced the likelihood of selecting products with these warnings.^20^

Cannabis packaging policies vary widely between the US and Canada and across US states. Canada mandates plain packaging with a white or black background, highly limited branding text or imagery, and a bright yellow warning label.^21^ Some US states have restrictions on youth-appealing packaging styles, but the specifics vary significantly from vague restrictions on all youth-appealing features to banning lists of specific features, such as cartoon characters, real or fictional humans, bright colors, and bubble fonts.^22^ In practice, however, audit studies have found a high prevalence of youth-appealing packaging and advertising on cannabis edibles and even copycat products mimicking popular food brands.^23,24,25,26^ Many US states prohibit unsubstantiated, false, or misleading health claims, but claims of relaxation, sedation, stress relief, and pain relief have been commonly seen on cannabis product packaging.^7,21,27,28,29^ Canada requires short, single-themed rotating warning messages, while most US states require composite health risk statements in a long paragraph.^21,30^

Empirical evidence on the effects of cannabis packaging policies has emerged recently, relying largely on experimental methods due to limited observational data on package exposure and health outcomes. Plain packaging received lower appeal ratings than branded packaging, was perceived to be more adult oriented, and was associated with lower purchase intentions and more accurate warning recall.^30,31,32,33,34^ Youth-appealing package features received longer visual attention and were preferred over normal branding features.^34,35^ Package marketing claims about relaxation were associated with increased intention to use cannabis.^33^ Package potency labels or visual indicators improved consumer knowledge about Δ^9^-tetrahydrocannabinol (THC) content and were preferred by consumers.^36,37^ Single-theme warnings were associated with increased warning recall compared with multitheme warnings.^31,38,39^ These studies broadly support the proposition that cannabis packaging features are associated with modified perceptions and behaviors among consumers.

In this study, we examined the association of packaging styles, health claims, and health warning themes with ability to recall health warnings as well as perceptions. We expect to make several unique contributions to literature and policymaking. (1) We designed a between-individuals full-factorial randomized experiment to establish strong causal inferences and allow for the estimation of the individual association of multiple simultaneously present packaging features. (2) We examined packaging styles, health claims, and health warning themes simultaneously to measure the relative importance of each package feature. (3) We focused on young adults aged 18 to 29 years, who are at the highest risk of using cannabis and developing adverse consequences.^4^ (4) We included young adults aged 18 to 20 years, who are prohibited from purchasing cannabis and tested differences between them and older age groups. (5) We recruited a large study sample and used quota-based sampling to make it representative of the population of interest.

## Methods

### Participants

From October 6 to 18, 2023, we recruited a convenience sample of 4500 participants from online panels through Qualtrics, a marketing company providing an online survey platform and recruitment services. The inclusion criteria were young adults aged 18 to 29 years residing in 1 of the 23 states or Washington, DC, with recreational cannabis legalized by the time of the survey. These jurisdictions regulate legal cannabis through policies, including packaging policies, so our study findings will be more policy relevant to these jurisdictions than to jurisdictions without legalization. Written informed consent was obtained when participants entered the online study. This study was approved by the Human Research Protections Program at the University of California, San Diego. This study followed the Strengthening the Reporting of Observational Studies in Epidemiology (STROBE) reporting guideline for cross-sectional observational studies.

The sample included two-thirds cannabis users, with cannabis use defined as using cannabis at least once in the past year regardless of administration method, and one-third nonusers. Although cannabis users were our primary population of interest due to their frequent exposure to cannabis packaging and greatest relevance to public health and policymaking, our inclusion of nonusers could provide insights into cannabis use initiation and relapse. For nonusers and users, we implemented demographic quotas based on age (18-20, 21-25, and 26-29 years), sex, and race and ethnicity (non-Hispanic White [hereafter, *White*] and non-Hispanic other [hereafter, *other*]) to make our convenience sample characteristics match with the nationally representative probabilistic sample characteristics in the 2021 National Survey on Drug Use and Health. Race and ethnicity were assessed in this study because they are the basic demographic variables frequently used in surveys that adopted the quota-matching method.

Qualtrics sent email invitations to their panelists who were likely to meet inclusion criteria based on their preexisting panelist profiles. Panelists completed screening questions and self-reported age, sex, race and ethnicity, state of residence, and cannabis use status before the start of the experiment to determine eligibility and demographic quotas. Recruitment continued until the target sample size of 4500 participants was achieved.

### Package Design

The packages involved in the experiment varied in 3 packaging features: packaging styles, health claims, and health warning themes. The 3 packaging styles included plain, normal branded, and youth-appealing packaging. The 3 health claims included none, pain relief, and sleep aid. The 7 warning themes included long-lasting effects, pregnancy-related harms, driving impairment, mental health problems, harms to youths, harms of high potency, and delayed effects. eTable 1 in Supplement 1 shows the full texts of all health warning themes. All the other product features were held constant in the experiment: 20 unflavored gummy edibles with 5 mg of THC per gummy. Gummies were chosen for the experiment because they accounted for more than 70% of the cannabis edible market share.^40^ Detailed information about how we selected and designed the 3 packaging features is provided in the eAppendix in Supplement 1.

### Experimental Procedure

The experimental procedure included an experiment involving viewing cannabis packaging with related outcome measures, survey questions on demographics and substance use, and a warning label recall task (a task asking participants to identify the warning label in the cannabis packaging image that they were previously shown in the packaging viewing experiment). All the elements were conducted online in a single session lasting a mean (SD) of 12.6 (8.2) minutes.

We used a between-individuals 3 (packaging styles) × 3 (health claims) × 7 (health warning themes) full-factorial randomized experiment. After receiving task instructions, participants were randomly assigned to 1 of the 63 possible packaging feature combinations and viewed the assigned packaging image for at least 10 seconds before the survey software allowed them to proceed. Participants then responded to several questions about the packaging while a small version of the same image appeared at the top of the screen for reference. An attention check question about the day of the week and a series of survey questions about demographic characteristics and substance use followed. At the end of the survey, participants answered a multiple-choice warning label recall question.

### Outcome Measures

Five outcomes were assessed, including warning recall, appeal rating, relative harm perception, adult-oriented appearance, and perceived target age group. Details about the measure constructs and scales are provided in eTable 2 in Supplement 1.

### Survey Questions About Individual Sociodemographic and Behavioral Variables

Survey questions gathered individual demographic and behavioral data, including age range (18-20, 21-25, and 26-29 years), sex, race and ethnicity (Hispanic, non-Hispanic Black [hereafter, *Black*], White, and other, which includes all remaining racial and ethnic categories that were not included in the first 3 categories), educational level (high school or less, some college, and bachelor’s degree or more), substance use (alcohol, cigarettes, and cannabis), and state of residence. These variables were all self-reported by participants and not further verified.

### Statistical Analysis

In the primary analyses, the associations of packaging styles, health claims, and health warning themes with outcomes were estimated with logit regressions for the binary outcome (warning recall) and ordered logit regressions for each ordered categorical outcome (appeal rating, relative harm perception, adult-oriented appearance, and perceived target age group). In all regressions, we controlled for the participant-level sociodemographic and behavioral variables. In the logit regression on warning recall, we additionally controlled for log (survey duration). This was because participants took varying amounts of time prior to the warning recall task and the survey duration may be associated with their ability to recall.

In the secondary analyses, the associations of potential moderators of age, cannabis use status, and cannabis use purposes were estimated by running regressions with interaction associations. These specifications were identical to our main specifications except for additional interaction terms between each packaging feature and (1) an indicator for ages 18 to 20 years, (2) an indicator for having used cannabis in the past year, and (3) an indicator for having ever used cannabis (this regression was conducted among past-year nonusers only).

As a robustness check, we excluded participants who incorrectly answered the attention check question and reran the main analyses. The SEs of all the regressions were clustered at the state level. The data had no missing values for all the variables used in the analysis. Data analyses were conducted in Stata SE, version 18.0 (StataCorp LLC). All *P* values were from 2-sided tests and results were deemed statistically significant at *P* < .05.

## Results

### Sample Characteristics

The Table reports descriptive statistics of the study sample of 4500 participants (2287 men [50.8%] and 2213 women [49.2%]; 1095 participants [24.3%] were aged 18-20 years, 1905 [42.3%] were aged 21-25 years, and 1500 [33.3%] were aged 26-29 years; 750 [16.7%] were Black, 982 [21.8%] were Hispanic, 2460 [54.7%] were White, and 308 [6.8%] were other race and ethnicity) by cannabis use status. eTable 3 in Supplement 1 compares the study sample with the 2021 National Survey on Drug Use and Health, from which we calculated age, sex, and race and ethnicity quotas.

**Table.** Descriptive Statistics of the Study Sample

Characteristic	No. (%)		
	All participants (N = 4500)	Cannabis nonusers (n = 1500)	Cannabis users (n = 3000)
Outcome measures, mean (SD)^a^			
Warning recall (binary outcome), %	52.9	59.9	49.4
Appeal rating (range, 0-10)	5.2 (2.9)	4.2 (2.8)	5.7 (2.7)
Relative harm perception (range, 1-5)	2.6 (1.1)	2.7 (1.0)	2.5 (1.1)
Adult-oriented appearance (range, 1-6)	3.7 (1.8)	3.4 (1.9)	3.9 (1.7)
Perceived target age group (range, 1-6)	3.2 (1.5)	3.0 (1.4)	3.3 (1.5)
Age, y			
18-20	1095 (24.3)	411 (27.4)	684 (22.8)
21-25	1905 (42.3)	572 (38.1)	1333 (44.4)
26-29	1500 (33.3)	517 (34.5)	983 (32.8)
Sex			
Male	2287 (50.8)	728 (48.5)	1559 (52.0)
Female	2213 (49.2)	772 (51.5)	1441 (48.0)
Race and ethnicity			
Hispanic	982 (21.8)	351 (23.4)	631 (21.0)
Non-Hispanic Black	750 (16.7)	234 (15.6)	516 (17.2)
Non-Hispanic White	2460 (54.7)	768 (51.2)	1692 (56.4)
Non-Hispanic other^b^	308 (6.8)	147 (9.8)	161 (5.4)
Educational level			
High school or less	1928 (42.8)	586 (39.1)	1342 (44.7)
Some college or associate’s degree	1585 (35.2)	536 (35.7)	1049 (35.0)
Bachelor’s degree or graduate degree	987 (21.9)	378 (25.2)	609 (20.3)
Substance use			
Past-year cannabis use	3000 (66.7)	0	3000 (100)
Medical-only cannabis use	NA	NA	448 (14.9)
Recreational-only cannabis use	NA	NA	1519 (50.6)
Dual-purpose cannabis use	NA	NA	1033 (34.4)
Past-month alcohol use	1933 (43.0)	481 (32.1)	1452 (48.4)
Past-month cigarette use	731 (16.2)	104 (6.9)	627 (20.9)
Observations, No.	4500	1500	3000

Abbreviation: NA, not applicable.

^a^ The SD is displayed for nonbinary outcome measures.

^b^ The non-Hispanic other category includes all other racial and ethnic identifications not included in Hispanic, non-Hispanic Black, and non-Hispanic White.

### Main Outcomes

The Figure displays the estimated odds ratios (ORs) for the 3 packaging features from the main analyses (see eTable 4 in Supplement 1 for details). Compared with normal branded packaging, plain packaging was associated with increased odds of correct warning recall (OR, 1.47 [95% CI, 1.27-1.70]), decreased appeal ratings (OR, 0.70 [95% CI, 0.61-0.80]), increased perceived relative harm (OR, 1.48 [95% CI, 1.27-1.74]), and increased adult-oriented appearance (OR, 1.34 [95% CI, 1.18-1.52]). Compared with normal branded packaging, youth-appealing packaging was associated with increased appeal ratings (OR, 1.40 [95% CI, 1.20-1.64]), decreased adult-oriented appearance (OR, 0.15 [95% CI, 0.13-0.18]), and decreased target age (OR, 0.19 [95% CI, 0.17-0.21]).

**Figure.**
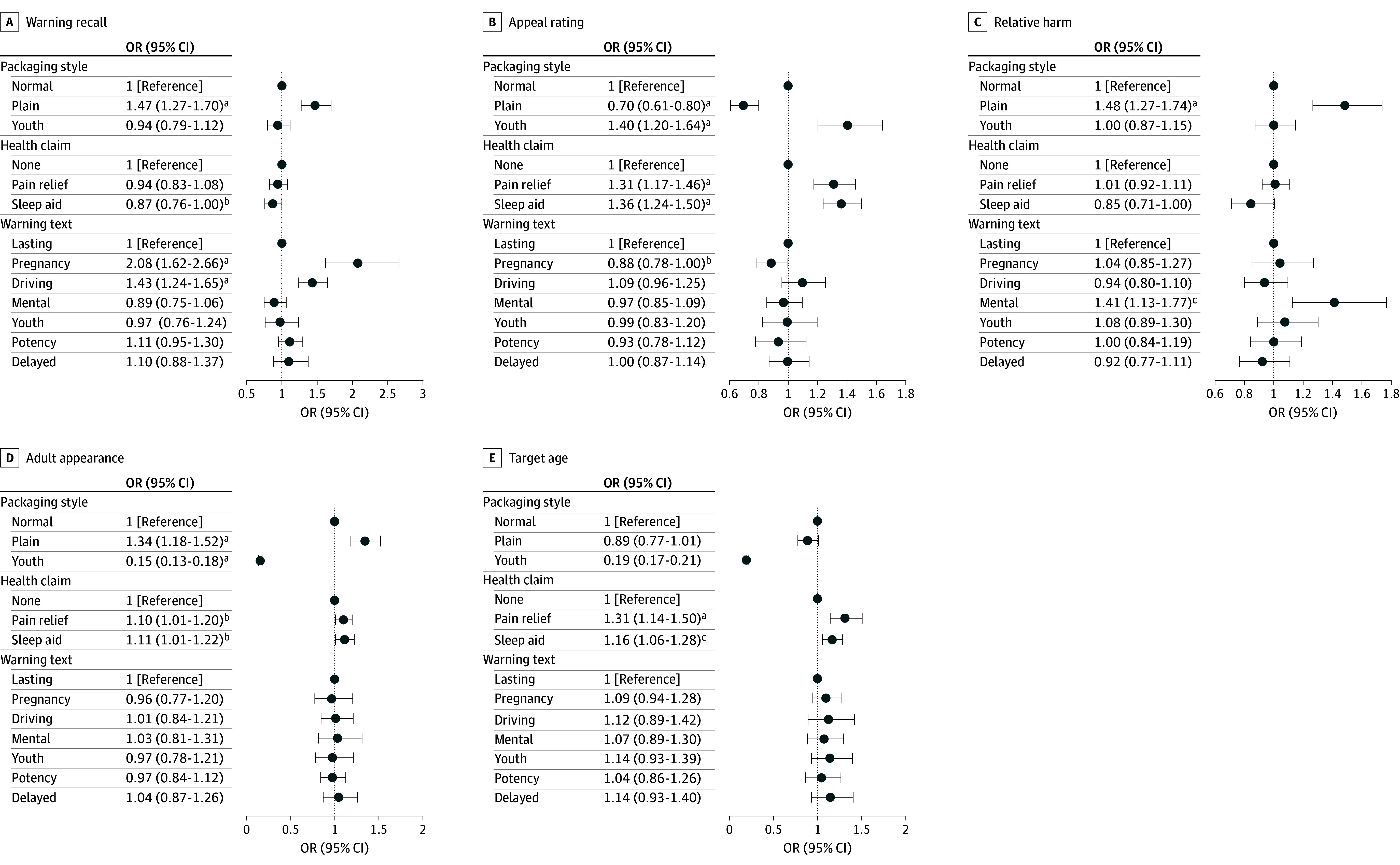
Main Associations of Packaging Features With Warning Recall and Perceptions Control variables included age, sex, race and ethnicity, educational level, substance use (alcohol, cigarettes, and cannabis), and state of residence. Regression on warning recall additionally controlled for log (survey duration in minutes). Standard errors were clustered at the state level. OR indicates odds ratio. ^a^*P* < .001. ^b^*P* < .05. ^c^*P* < .01.

Compared with no health claim, the pain relief and sleep aid claims were both associated with increased appeal ratings (pain relief: OR, 1.31 [95% CI, 1.17-1.46]; sleep aid: OR, 1.36 [95% CI, 1.24-1.50]) and increased target age (pain relief: OR, 1.31 [95% CI, 1.14-1.50]; sleep aid: OR, 1.16 [95% CI, 1.06-1.28]) (eTable 4 in Supplement 1). Compared with the health warning about long-lasting effects, warnings about pregnancy harms and driving impairment were both associated with increased odds of correct warning recall (pregnancy harms: OR, 2.08 [95% CI, 1.62-2.66]; driving impairment: OR, 1.43 [95% CI, 1.24-1.65]).

### Interaction Associations

eFigure 1 in Supplement 1 displays interaction associations between participants aged 18 to 20 years and packaging features (see eTable 5 in Supplement 1 for details). Compared with normal branded packaging, youth-appealing packaging was associated with higher appeal ratings among participants aged 18 to 20 years than among older participants (OR, 1.38 [95% CI, 1.03-1.86]).

eFigure 2 in Supplement 1 displays interaction associations between cannabis use status and packaging features (see eTable 6 in Supplement 1 for details). Compared with packaging without health claims, packaging with either pain relief or sleep aid claims were associated with lower appeal ratings among users than nonusers (pain relief: OR, 0.71 [95% CI, 0.59-0.86]; sleep aid: OR, 0.76 [95% CI, 0.59-0.99]).

eFigure 3 in Supplement 1 (see eTable 7 in Supplement 1 for details) displays interaction associations between former cannabis use and packaging features among past-year nonusers. Compared with normal branded packaging, both plain and youth-appealing packaging were associated with lower odds of correct warning recall among former users than never users (plain packaging: OR, 0.58 [95% CI, 0.36-0.93]; youth-appealing packaging: OR, 0.57 [95% CI, 0.39-0.83]).

### Robustness Check

We removed 276 participants (6.1% of the study sample) who incorrectly answered the attention check question. No substantive changes were found for the main results.

## Discussion

Our study provided several policy-relevant findings on the association of cannabis packaging features with health warning recall and perceptions. First, plain packaging was associated with increased odds of correct warning recall, decreased appeal ratings, and increased perceived harm. These findings are in line with the emerging literature on cannabis packaging, which found that plain packaging was associated with decreased appeal ratings, decreased purchase intentions, and increased warning recall for certain warning themes.^30,31,32,33^ They also echo the literature on tobacco control, which suggested that plain tobacco packaging increased visual attention to warnings, decreased product appeal, and decreased misperceptions of harms.^14^ The findings imply that applying Canada’s plain packaging requirements to products in US states has the potential to increase comprehension and recall of warnings, reduce product appeal, and more closely align perceived health risks with actual risks among young adults.

Second, our results suggested that youth-appealing packaging was associated with increased appeal ratings, even while the product was perceived as looking more childish and targeting younger age groups. Our interaction associations analyses further showed that this appeal rating association was stronger among participants aged 18 to 20 years than among older participants, suggesting that youth-appealing packaging had stronger associations with appeal among the younger population. These findings support our previous work, which found that similar youth-appealing packaging was preferred over normal branded packaging.^34^ These findings on youth appeal may be concerning to public health policymakers, as young people experience more severe health harms from problematic cannabis use.^5,6^ If plain packaging is not politically or practically feasible in the US, policymakers in the US may instead consider comprehensive bans on youth-appealing packaging features to improve public health outcomes. Strict enforcement would be essential for the success of such a policy because youth-appealing features have been frequently observed even in places already with such bans.^23,24,25,26^

Third, both pain relief and sleep aid claims were associated with increased appeal ratings. This finding is related to previous research, which found that marketing claims such as “helps you relax” on packaging were associated with increased intentions to use cannabis edibles.^33^ Although unsubstantiated health claims are prohibited in several states, they are neither universal nor consistent.^7,27^ The interaction associations analyses further suggested that, although health claims were associated with higher appeal ratings for both cannabis users and nonusers, health claims had a stronger association with appeal ratings for nonusers, who may have limited knowledge on the therapeutic benefits and health harms associated with cannabis use.

Fourth, we found that health warnings about pregnancy harm and driving impairment were associated with increased odds of correct warning recall. This finding could be because these 2 themes are inherently more salient, concerning, or credible to people or because cannabis harms associated with these 2 themes have been more widespread than others. Related research has found that warnings on driving impairment were most believable, while warnings on addiction and negative psychiatric outcomes were the least believable.^41^ Health warning themes had limited associations with other outcomes in our study, particularly harm perceptions, calling for future research to improve existing health warnings for more effective communication.

### Limitations

Our study has several limitations. First, our outcomes included only warning label recall and subjective perceptions, which were not directly associated with cannabis purchase patterns or consumption behaviors. This limitation was inevitable because of the cross-sectional study design and self-reporting of the measures. However, these outcomes have been commonly adopted in the tobacco and cannabis literature and are associated with behavioral outcomes such as smoking cessation attempts.^11,14^ Second, not all policy-relevant packaging features were examined due to the full-factorial experimental design. We selected packaging features based on public health effect and novelty in the literature. Some promising features to study in future research include single-theme vs multitheme warnings, various styles of pictorial warnings, and warning label placement and size. Third, we used gummy edibles due to their popularity and common presence of youth-appealing features on packaging. The findings, however, may not extend to other product types, such as flowers, concentrates, and vaping devices. Fourth, we designed the packaging based on popular products, but the findings may not generalize to package designs with different visual features. Fifth, we used a quota-based convenience sampling approach instead of probabilistic sampling due to cost constraints. Our sample may not be representative of other characteristics not matched by quotas (such as educational level) or of more granular category breakdowns than we implemented (such as more detailed categories of race and ethnicity). Our findings may not generalize to populations younger than 18 years of age or older than 29 years of age, in US states without recreational cannabis legalization, or outside the US.

## Conclusions

In this cross-sectional study using a between-individual full-factorial experiment varying packaging styles, health claims, and health warning themes of edible cannabis products, the findings suggested that packaging policies requiring plain packaging, prohibiting youth-appealing features, regulating unsubstantiated health claims, and refining warning messages may have potential to improve health warning recall and accurate harm perceptions of cannabis edible products among young adults. Future observational studies evaluating the effects of these regulations are recommended.

## Notes

Supplement 1. **eAppendix.** Package Image Design **eFigure 1.** Interaction Effects Between Age 18-20 and Package Features **eFigure 2.** Interaction Effects Between Past-Year Cannabis Use and Package Features **eFigure 3.** Interaction Effects Between Lifetime Cannabis Use and Package Features Among Past-Year Nonusers **eTable 1.** Full Texts of Health Warning Themes **eTable 2.** Outcome Measure Details **eTable 3.** Comparison of Demographics Between Study Sample and the 2021 National Survey on Drug Use and Health (NSDUH) **eTable 4.** Main Effects of Package Features on Warning Recall and Perceptions **eTable 5.** Interaction Effects Between Age 18-20 and Package Features **eTable 6.** Interaction Effects Between Cannabis Use Status and Package Features **eTable 7.** Interaction Effects Between Lifetime Cannabis Use and Package Features Among Past-Year Nonusers
eReferences.

Supplement 2.
Data Sharing Statement
