# Supplementary material for: Packaging of Cannabis Edibles, Health Warning Recall, and Perceptions Among Young Adults
Source: JAMA Netw Open. 2025 Apr 3;8(4):e253117. doi: 10.1001/jamanetworkopen.2025.3117 (PMC11969283; doi:10.1001/jamanetworkopen.2025.3117)
Supplement: Supplement 1. — eAppendix. Package Image Design eFigure 1. Interaction Effects Between Age 18-20 and Package Features eFigure 2. Interaction Effects Between Past-Year Cannabis Use and Package Features eFigure 3. Interaction Effects Between Lifetime Cannabis Use and Package Features Among Past-Year Nonusers eTable 1. Full Texts of Health Warning Themes eTable 2. Outcome Measure Details eTable 3. Comparison of Demographics Between Study Sample and the 2021 National Survey on Drug Use and Health (NSDUH) eTable 4. Main Effects of Package Features on Warning Recall and Perceptions eTable 5. Interaction Effects Between Age 18-20 and Package Features eTable 6. Interaction Effects Between Cannabis Use Status and Package Features eTable 7. Interaction Effects Between Lifetime Cannabis Use and Package Features Among Past-Year Nonusers eReferences. [file jamanetwopen-e253117-s001.pdf]

Supplementary Online Content

Cooper M, Shi Y. Packaging of cannabis edibles, health warning recall, and perceptions among young adults. *JAMA Netw Open.* 2025;8(4):e253117.
doi:10.1001/jamanetworkopen.2025.3117

eAppendix. Package Image Design

eFigure 1. Interaction Effects Between Age 18-20 and Package Features

eFigure 2. Interaction Effects Between Past-Year Cannabis Use and Package Features

eFigure 3. Interaction Effects Between Lifetime Cannabis Use and Package Features Among Past-Year Nonusers

eTable 1. Full Texts of Health Warning Themes

eTable 2. Outcome Measure Details

eTable 3. Comparison of Demographics Between Study Sample and the 2021 National Survey on Drug Use and Health (NSDUH)

eTable 4. Main Effects of Package Features on Warning Recall and Perceptions

eTable 5. Interaction Effects Between Age 18-20 and Package Features

eTable 6. Interaction Effects Between Past-Year Cannabis Use and Package Features

eTable 7. Interaction Effects Between Lifetime Cannabis Use and Package Features Among Past-Year Nonusers

eReferences.

This supplementary material has been provided by the authors to give readers additional information about their work.

eAppendix. Package Image Design

We worked with a professional graphic designer to develop package images, which were modified from our previous research.[1,2] The plain package style was based on the requirements for recreational cannabis sold in Canada: a plain white background with minimal branding text, no logos or graphics, and a bright yellow warning label. The normal branded package style was closely based on a real cannabis gummy edible package sold in the U.S. This package was selected for replication due to its popularity, visual similarity to other packages, use of neutral colors, and segmented package regions to allow the placement of varied package features. We selected two youth-appealing features to include in the youth-appealing package based on several published papers measuring the prevalence of youth-appealing cannabis packaging:[3-6] a cartoon character instead of a logo, and colorful bubble font instead of regular font for the brand name. These two features were replaced on the normal branded package, holding constant their position and size and keeping the rest of the package image identical.

The health claims of "Pain Relief" and "Sleep Aid" were selected based on health benefits frequently described on cannabis packages, websites, and marketing materials.[7,8]

The seven health warning themes were identical to the rotating warning messages required in Canada (eTable 1). We omitted two of their messages that were relevant only to smoking and topical products. The themes of these warnings have been commonly covered in U.S. state warnings as well, with the most common themes being pregnancy-related harms, driving impairment, keeping cannabis away from children, and delayed intoxication.[9] For example, the required cannabis warning label in California is as follows: "Government warning: This package contains cannabis, a Schedule 1 controlled substance. Keep out of reach of children and animals. Cannabis may only be possessed or consumed by persons 21 years of age or older unless the person is a qualified patient. Cannabis use while pregnant or breastfeeding may be harmful. Consumption of cannabis impairs your ability to drive and operate machinery. Please use extreme caution."

© 2025 Cooper M et al. *JAMA Network Open.*

eFigure 1: Interaction Effects between Age 18-20 and Package Features

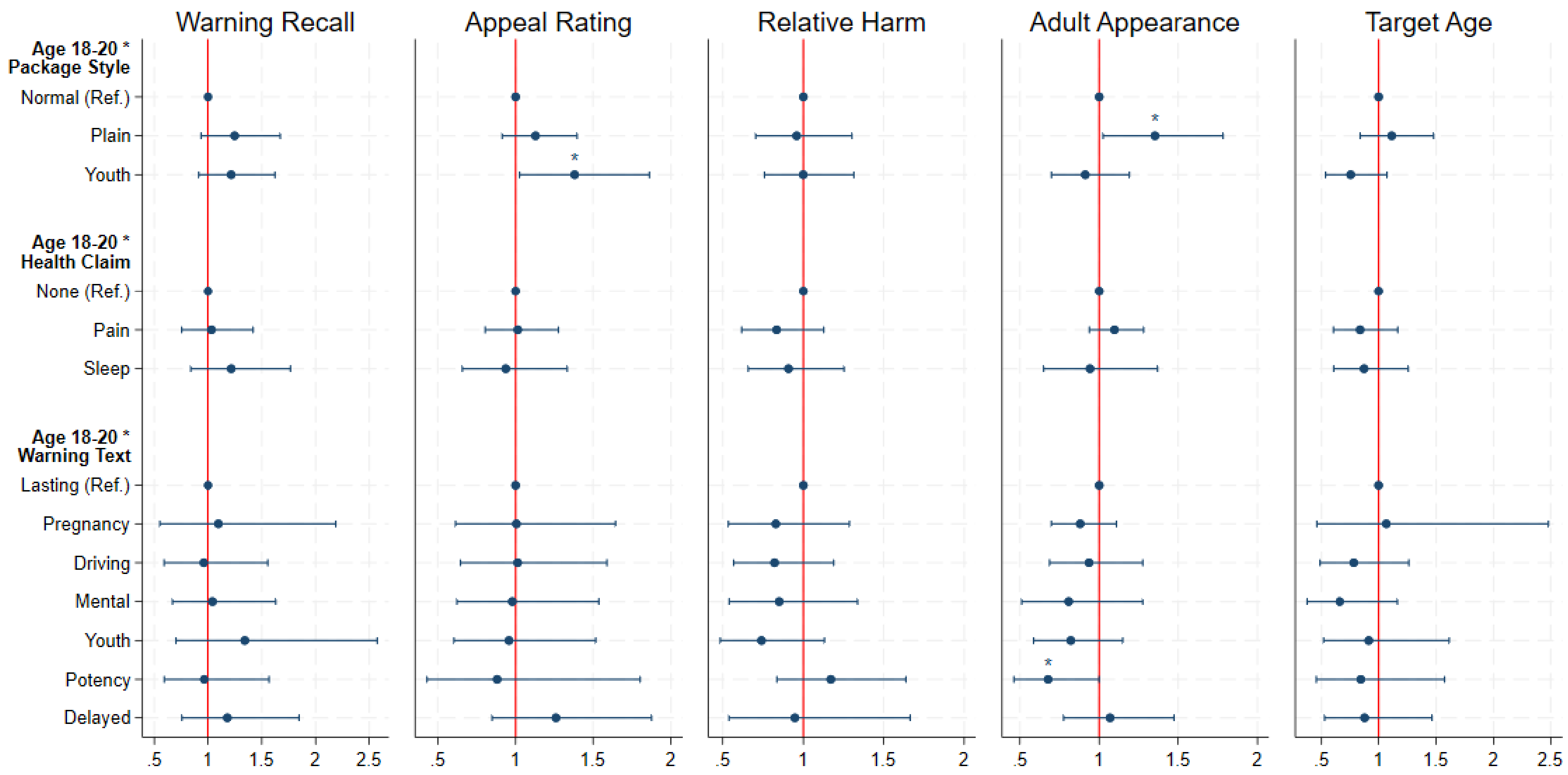

Figure Notes: *P<.05, **P<.01, ***P<.001. The figure displays interaction effects between the listed package feature and an indicator for age 18-20. The regression specifications are identical to the main effects specification except for these additional interaction effects. Odds ratios are displayed with 95% confidence intervals in brackets. Control variables included are age, sex, race/ethnicity, education, substance use (alcohol, cigarettes, and cannabis), and state of residence. Regression on warning recall additionally controlled for log(survey duration in minutes). Standard errors were clustered at the state level.

© 2025 Cooper M et al. *JAMA Network Open.*

eFigure 2: Interaction Effects between Past-Year Cannabis Use and Package Features

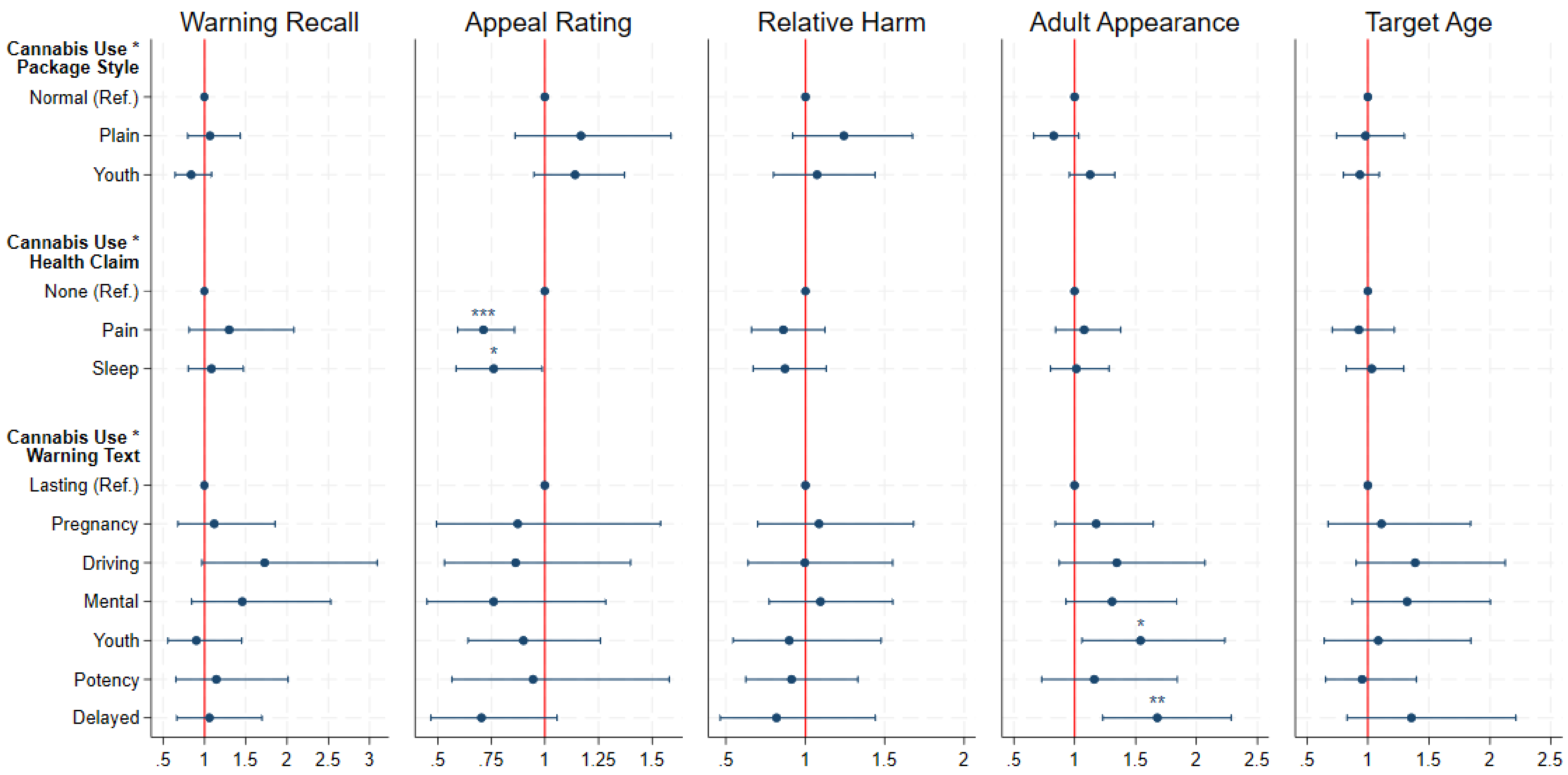

Figure Notes: *P<.05, **P<.01, ***P<.001. The figure displays interaction effects between the listed package feature and past-year cannabis use. The regression specifications are identical to the main effects specification except for these additional interaction effects. Odds ratios are displayed with 95% confidence intervals in brackets. Control variables included are age, sex, race/ethnicity, education, substance use (alcohol, cigarettes, and cannabis), and state of residence. Regression on warning recall additionally controlled for log(survey duration in minutes). Standard errors were clustered at the state level.

© 2025 Cooper M et al. *JAMA Network Open.*

eFigure 3. Interaction Effects Between Lifetime Cannabis Use and Package Features Among Past-Year Nonusers

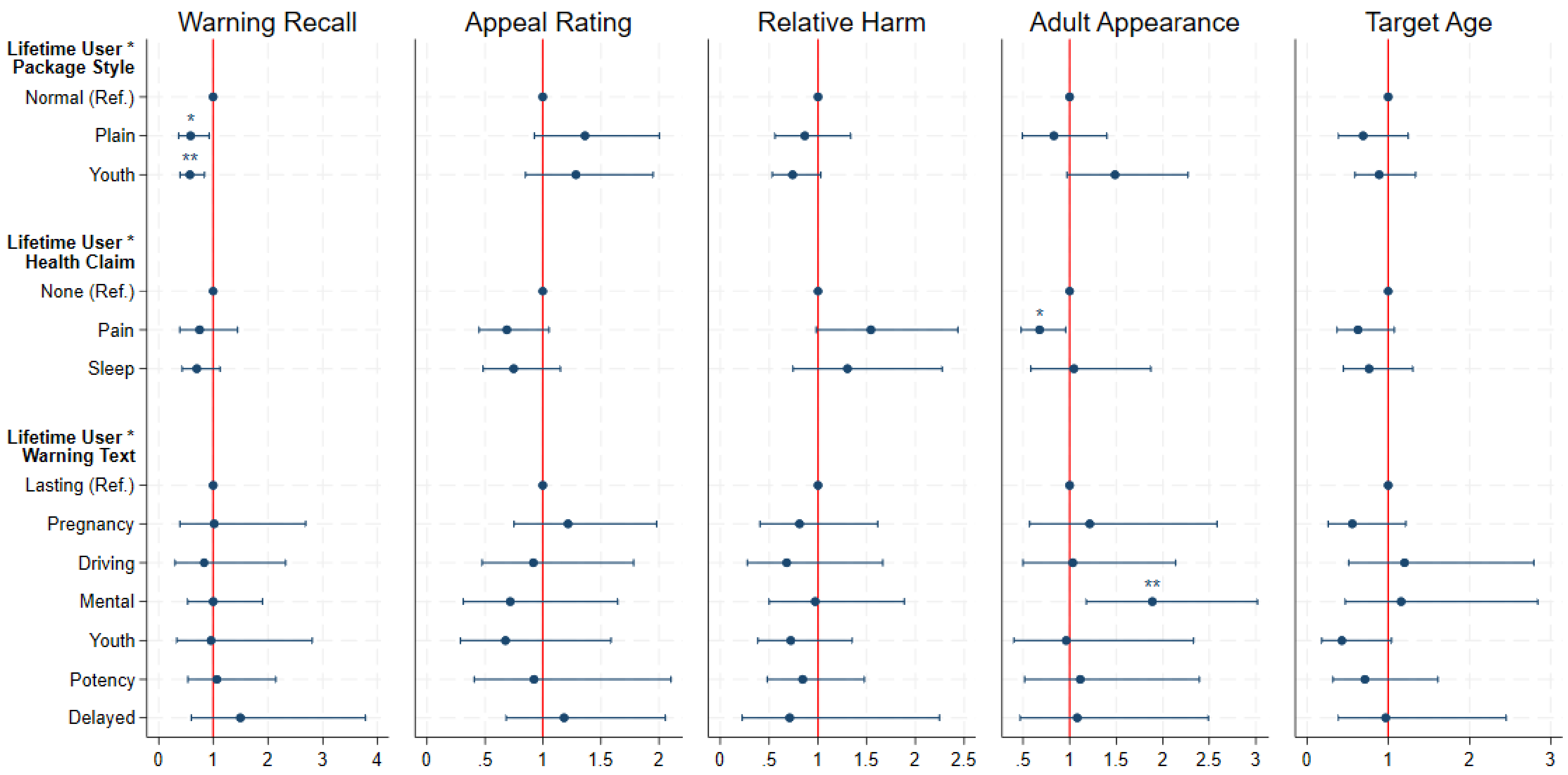

Figure Notes: *P<.05, **P<.01, ***P<.001. The figure displays interaction effects between the listed package feature and lifetime cannabis use, only among past-year nonusers. The regression specifications were identical to the main effects specification except for these additional interaction effects. Odds ratios are displayed with 95% confidence intervals in brackets. Control variables included age, sex, race/ethnicity, education, substance use (alcohol, cigarettes, and cannabis), and state of residence. Regression on warning recall additionally controlled for log(survey duration in minutes). Standard errors were clustered at the state level.

© 2025 Cooper M et al. *JAMA Network Open.*

eTable 1. Full Texts of Health Warning Themes

Warning Label Title	Full Text
“Lasting”	**WARNING: The effects from eating or drinking cannabis can be long-lasting.** The effects can last between 6 and 12 hours following use.
“Pregnancy”	**WARNING: Do not use if pregnant or breast feeding.** Substances in cannabis are transferred from the mother to child and can harm your baby.
“Driving”	**WARNING: Do not drive or operate heavy equipment after using cannabis.** Cannabis can cause drowsiness and impair your ability to concentrate and make quick decisions.
“Mental”	**WARNING: Frequent and prolonged use of cannabis containing THC can contribute to mental health problems over time.** Daily or near-daily use increases the risk of dependence and may bring on or worsen disorders related to anxiety and depression.
“Youth”	**WARNING: Adolescents and young adults are at greater risk of harms from cannabis.** Daily or near-daily use over a prolonged period of time can harm brain development and function.
“Potency”	**WARNING: The higher the THC content of a product, the more likely you are to experience adverse effects and greater levels of impairment.** THC can cause anxiety and impair memory and concentration.
“Delayed”	**WARNING: It can take up to 4 hours to feel the full effects from eating or drinking cannabis.** Consuming more within this time period can result in adverse effects that may require medical attention.

© 2025 Cooper M et al. *JAMA Network Open.*

eTable 2. Outcome Measure Details

**Outcome Measure**	**Measure Details**
Warning recall[9,10]	Binary outcome that takes value of 1 if a participant correctly selects the warning label they were shown in a multiple choice question displaying all 7 possible warnings in the study.
Appeal rating[2,11-13]	"How appealing would this product be to try?" Assessed on a scale from 0 ("not at all appealing") to 10 ("extremely appealing").
Relative harm[13,14]	"Compared to other marijuana, would you say the marijuana in this package is…" Assessed on a scale from 1 ("a lot less harmful") to 5 ("a lot more harmful").
Adult-oriented appearance[13]	"Do you think this marijuana package looks…" Assessed on a scale from 0 ("childish") to 6 ("grown-up").
Perceived target age group[11,15]	"In your opinion, what age group would be most likely to try this product?" Assessed with ordered categories: 1 (12-17 years old), 2 (18-20 years old), 3 (21-25 years old), 4 (26-29 years old), 5 (30-34 years old), 6 (35+ years old).

© 2025 Cooper M et al. *JAMA Network Open.*

eTable 3. Comparison of Demographics Between Study Sample and the 2021 National Survey on Drug Use and Health (NSDUH)

	Cannabis Nonusers			Cannabis Users		
	Study Sample	NSDUH Sample		Study Sample	NSDUH Sample	
	%		p-value of Chi-square test	%		p-value of Chi-square test
**Age:**						
18-20	27.40	27.41		22.80	22.80	
21-25	38.13	38.14	1.00	44.43	44.44	1.00
26-29	34.47	34.45		32.77	32.77	
**Sex:**						
Male	48.53	48.53	1.00	51.97	51.96	1.00
Female	51.47	51.47		48.03	48.04	
**Race & Ethnicity:**						
Hispanic	23.40	24.21		21.03	19.68	
Non-Hispanic Black	15.60	13.73	0.16	17.20	14.92	0.00
Non-Hispanic White	51.20	51.19		56.40	56.41	
Non-Hispanic Other	9.80	10.88		5.37	8.99	
**Education:**						
High School or Less	39.07	40.79		44.73	38.62	
Some College	35.73	37.10	0.03	34.97	39.29	0.00
Bachelor's Degree or More	25.20	22.11		20.30	22.09	
Observations	1,500	12,047		3,000	5,997	

Table Notes: NSDUH sample included cannabis nonusers and users aged 18-29. Analytical weights were used in the NSDUH sample statistics. Quotas were implemented only on age, sex, and race/ethnicity (non-Hispanic white or other minorities).

© 2025 Cooper M et al. *JAMA Network Open.*

eTable 4. Main Effects of Package Features on Warning Recall and Perceptions

	Warning Recall	Appeal Rating	Relative Harm	Adult Appearance	Target Age
	Odds Ratio [95% CI]				
**Package Style:**					
Normal	Ref.	Ref.	Ref.	Ref.	Ref.
Plain	1.47*** [1.27, 1.70]	0.70*** [0.61, 0.80]	1.48*** [1.27, 1.74]	1.34*** [1.18, 1.52]	0.89 [0.77, 1.01]
Youth-Appealing	0.94 [0.79, 1.12]	1.40*** [1.20, 1.64]	1.00 [0.87, 1.15]	0.15*** [0.13, 0.18]	0.19*** [0.17, 0.21]
**Health Claim:**					
None	Ref.	Ref.	Ref.	Ref.	Ref.
Pain	0.94 [0.83, 1.08]	1.31*** [1.17, 1.46]	1.01 [0.92, 1.11]	1.10* [1.01, 1.20]	1.31*** [1.14, 1.50]
Sleep	0.87* [0.76, 1.00]	1.36*** [1.24, 1.50]	0.85 [0.71, 1.00]	1.11* [1.01, 1.22]	1.16** [1.06, 1.28]
**Warning Text:**					
Lasting	Ref.	Ref.	Ref.	Ref.	Ref.
Pregnancy	2.08*** [1.62, 2.66]	0.88* [0.78, 1.00]	1.04 [0.85, 1.27]	0.96 [0.77, 1.20]	1.09 [0.94, 1.28]
Driving	1.43*** [1.24, 1.65]	1.09 [0.96, 1.25]	0.94 [0.80, 1.10]	1.01 [0.84, 1.21]	1.12 [0.89, 1.42]
Mental	0.89 [0.75, 1.06]	0.97 [0.85, 1.09]	1.41** [1.13, 1.77]	1.03 [0.81, 1.31]	1.07 [0.89, 1.30]
Youth	0.97 [0.76, 1.24]	0.99 [0.83, 1.20]	1.08 [0.89, 1.30]	0.97 [0.78, 1.21]	1.14 [0.93, 1.39]
Potency	1.11 [0.95, 1.30]	0.93 [0.78, 1.12]	1.00 [0.84, 1.19]	0.97 [0.84, 1.12]	1.04 [0.86, 1.26]
Delayed	1.10 [0.88, 1.37]	1.00 [0.87, 1.14]	0.92 [0.77, 1.11]	1.04 [0.87, 1.26]	1.14 [0.93, 1.40]
**Age:**					
18-20	1.13 [0.90, 1.41]	0.74*** [0.63, 0.88]	1.48*** [1.32, 1.65]	0.94 [0.77, 1.15]	0.42*** [0.34, 0.51]
21-25	0.93 [0.77, 1.14]	0.82** [0.72, 0.94]	1.20** [1.06, 1.35]	0.91 [0.79, 1.03]	0.57*** [0.51, 0.63]
26-29	Ref.	Ref.	Ref.	Ref.	Ref.
**Sex:**					
Female	Ref.	Ref.	Ref.	Ref.	Ref.
Male	0.69*** [0.61, 0.79]	1.54*** [1.32, 1.79]	1.14** [1.05, 1.24]	1.44*** [1.34, 1.54]	1.12 [1.00, 1.25]

© 2025 Cooper M et al. *JAMA Network Open.*

**Race & Ethnicity:**					
Non-Hispanic White	Ref.	Ref.	Ref.	Ref.	Ref.
Hispanic	0.80** [0.68, 0.95]	1.19* [1.03, 1.37]	1.13* [1.03, 1.25]	0.92 [0.82, 1.04]	0.68*** [0.62, 0.75]
Non-Hispanic Black	0.55*** [0.48, 0.63]	1.24** [1.06, 1.46]	1.09 [0.90, 1.32]	0.88 [0.74, 1.03]	0.72*** [0.62, 0.84]
Non-Hispanic Other Minority	0.75 [0.53, 1.05]	1.00 [0.78, 1.28]	1.03 [0.89, 1.19]	0.77* [0.60, 1.00]	0.73* [0.53, 0.99]
**Education:**					
High School or Less	Ref.	Ref.	Ref.	Ref.	Ref.
Some College or Associate Degree	1.12 [0.95, 1.32]	1.03 [0.91, 1.16]	1.20** [1.07, 1.34]	0.91 [0.79, 1.05]	1.17** [1.05, 1.29]
Bachelor's Degree or Graduate Degree	1.09 [0.94, 1.26]	1.05 [0.92, 1.20]	1.70*** [1.44, 2.02]	0.84* [0.72, 0.96]	1.10 [0.96, 1.26]
**Substance Use:**					
Past-Year Cannabis Use	0.61*** [0.51, 0.72]	2.40*** [2.06, 2.81]	0.73*** [0.65, 0.82]	1.47*** [1.31, 1.65]	1.47*** [1.30, 1.65]
Past-Month Alcohol Use	1.55*** [1.30, 1.84]	0.84* [0.74, 0.96]	0.74*** [0.64, 0.86]	1.03 [0.93, 1.14]	1.25*** [1.15, 1.36]
Past-Month Cigarette Use	0.80** [0.70, 0.93]	1.57*** [1.32, 1.88]	0.91 [0.77, 1.08]	1.14 [0.95, 1.37]	0.92 [0.81, 1.05]
**Survey Behavior:**					
Log Survey Duration (Minutes)	1.12 [1.00, 1.27]				
Observations	4,500	4,500	4,500	4,500	4,500

Table Notes: *P<.05, **P<.01, ***P<.001. Regressions additionally controlled for state of residence (suppressed from table). Standard errors were clustered at the state level.

© 2025 Cooper M et al. *JAMA Network Open.*

eTable 5. Interaction Effects Between Age 18-20 and Package Features

	**Warning Recall**	**Appeal Rating**	**Relative Harm**	**Adult Appearance**	**Target Age**
	Odds Ratio [95% CI]				
**Age 18-20 * Package Style**					
Normal	Ref.	Ref.	Ref.	Ref.	Ref.
Plain	1.25 [0.93, 1.67]	1.13 [0.91, 1.40]	0.96 [0.70, 1.30]	1.35* [1.02, 1.78]	1.11 [0.84, 1.48]
Youth-Appealing	1.22 [0.91, 1.62]	1.38* [1.03, 1.86]	1.00 [0.76, 1.32]	0.91 [0.70, 1.19]	0.76 [0.53, 1.07]
**Age 18-20 * Health Claim**					
None	Ref.	Ref.	Ref.	Ref.	Ref.
Pain	1.03 [0.75, 1.42]	1.01 [0.80, 1.28]	0.83 [0.62, 1.13]	1.10 [0.94, 1.28]	0.84 [0.60, 1.17]
Sleep	1.22 [0.84, 1.77]	0.94 [0.66, 1.33]	0.91 [0.66, 1.25]	0.94 [0.65, 1.37]	0.87 [0.61, 1.26]
**Age 18-20 * Warning Text**					
Lasting	Ref.	Ref.	Ref.	Ref.	Ref.
Pregnancy	1.10 [0.55, 2.19]	1.01 [0.61, 1.64]	0.83 [0.53, 1.29]	0.88 [0.70, 1.11]	1.07 [0.46, 2.48]
Driving	0.96 [0.59, 1.56]	1.01 [0.65, 1.59]	0.82 [0.57, 1.19]	0.94 [0.69, 1.27]	0.78 [0.49, 1.26]
Mental Health	1.04 [0.67, 1.63]	0.98 [0.62, 1.54]	0.85 [0.54, 1.34]	0.81 [0.51, 1.27]	0.66 [0.37, 1.16]
Youth	1.34 [0.70, 2.58]	0.96 [0.60, 1.52]	0.74 [0.48, 1.13]	0.82 [0.59, 1.15]	0.91 [0.52, 1.61]
Potency	0.96 [0.59, 1.57]	0.88 [0.43, 1.80]	1.17 [0.84, 1.64]	0.68* [0.46, 1.00]	0.85 [0.45, 1.57]
Delayed	1.18 [0.76, 1.85]	1.26 [0.85, 1.88]	0.95 [0.54, 1.67]	1.07 [0.78, 1.47]	0.88 [0.53, 1.46]
Observations	4,500	4,500	4,500	4,500	4,500

Table Notes: *P<.05, **P<.01, ***P<.001. The model specifications were identical to the main effects regressions, except that the interaction effects between an indicator for age 18-20 and package features were added. Only interaction effects are displayed; other coefficients are estimated but suppressed from the table. Standard errors were clustered at the state level.

© 2025 Cooper M et al. *JAMA Network Open.*

eTable 6. Interaction Effects Between Past-Year Cannabis Use and Package Features

	Warning Recall	Appeal Rating	Relative Harm	Adult Appearance	Target Age
	Odds Ratio [95% CI]				
**Cannabis Use * Package Style**					
Normal	Ref.	Ref.	Ref.	Ref.	Ref.
Plain	1.07 [0.80, 1.44]	1.17 [0.86, 1.59]	1.24 [0.92, 1.68]	0.83 [0.67, 1.03]	0.98 [0.74, 1.30]
Youth-Appealing	0.84 [0.64, 1.09]	1.14 [0.95, 1.37]	1.07 [0.80, 1.44]	1.13 [0.96, 1.33]	0.94 [0.80, 1.09]
**Cannabis Use * Health Claim**					
None	Ref.	Ref.	Ref.	Ref.	Ref.
Pain	1.30 [0.81, 2.08]	0.71*** [0.59, 0.86]	0.86 [0.66, 1.12]	1.08 [0.84, 1.38]	0.93 [0.71, 1.22]
Sleep	1.09 [0.80, 1.47]	0.76* [0.59, 0.99]	0.87 [0.67, 1.13]	1.01 [0.80, 1.28]	1.03 [0.82, 1.29]
**Cannabis Use * Warning Text**					
Lasting	Ref.	Ref.	Ref.	Ref.	Ref.
Pregnancy	1.12 [0.68, 1.86]	0.87 [0.49, 1.54]	1.08 [0.70, 1.68]	1.18 [0.84, 1.64]	1.11 [0.67, 1.84]
Driving	1.73 [0.97, 3.09]	0.86 [0.53, 1.40]	1.00 [0.64, 1.55]	1.35 [0.87, 2.07]	1.39 [0.90, 2.13]
Mental Health	1.46 [0.84, 2.53]	0.76 [0.45, 1.28]	1.09 [0.77, 1.55]	1.31 [0.93, 1.84]	1.32 [0.87, 2.00]
Youth	0.90 [0.56, 1.45]	0.90 [0.64, 1.26]	0.90 [0.55, 1.48]	1.54* [1.06, 2.23]	1.09 [0.64, 1.84]
Potency	1.15 [0.65, 2.01]	0.95 [0.57, 1.58]	0.91 [0.63, 1.33]	1.16 [0.73, 1.84]	0.95 [0.65, 1.40]
Delayed	1.06 [0.67, 1.70]	0.70 [0.47, 1.06]	0.82 [0.46, 1.44]	1.68** [1.23, 2.29]	1.36 [0.83, 2.22]
Observations	4,500	4,500	4,500	4,500	4,500

Table Notes: *P<.05, **P<.01, ***P<.001. The model specifications were identical to the main effects regressions, except that the interaction effects between past-year cannabis use and package features were added to each specification. Only interaction effects are displayed; other coefficients are estimated but suppressed from the table. Standard errors were clustered at the state level.

eTable 7. Interaction Effects Between Lifetime Cannabis Use and Package Features Among Past-Year Nonusers

	Warning Recall	Appeal Rating	Relative Harm	Adult Appearance	Target Age
	Odds Ratio [95% CI]				
**Lifetime Use * Package Style**					
Normal	Ref.	Ref.	Ref.	Ref.	Ref.
Plain	0.58* [0.36, 0.93]	1.36 [0.93, 2.00]	0.86 [0.56, 1.33]	0.83 [0.49, 1.40]	0.69 [0.39, 1.25]
Youth-Appealing	0.57** [0.39, 0.83]	1.29 [0.85, 1.95]	0.74 [0.53, 1.03]	1.49 [0.97, 2.27]	0.89 [0.59, 1.34]
**Lifetime Use * Health Claim**					
None	Ref.	Ref.	Ref.	Ref.	Ref.
Pain	0.75 [0.38, 1.44]	0.69 [0.45, 1.05]	1.55 [0.98, 2.44]	0.68* [0.48, 0.96]	0.63 [0.37, 1.08]
Sleep	0.69 [0.43, 1.13]	0.75 [0.48, 1.15]	1.30 [0.74, 2.28]	1.05 [0.58, 1.87]	0.77 [0.45, 1.30]
**Lifetime Use * Warning Text**					
Lasting	Ref.	Ref.	Ref.	Ref.	Ref.
Pregnancy	1.02 [0.39, 2.69]	1.22 [0.75, 1.98]	0.81 [0.41, 1.62]	1.22 [0.57, 2.58]	0.56 [0.26, 1.22]
Driving	0.83 [0.30, 2.32]	0.92 [0.47, 1.78]	0.68 [0.28, 1.67]	1.03 [0.50, 2.14]	1.20 [0.52, 2.79]
Mental Health	1.00 [0.53, 1.90]	0.72 [0.31, 1.64]	0.97 [0.50, 1.89]	1.89** [1.18, 3.02]	1.16 [0.47, 2.84]
Youth	0.96 [0.33, 2.81]	0.68 [0.29, 1.59]	0.72 [0.39, 1.35]	0.96 [0.40, 2.33]	0.43 [0.18, 1.04]
Potency	1.07 [0.53, 2.14]	0.92 [0.41, 2.10]	0.84 [0.48, 1.48]	1.11 [0.52, 2.40]	0.71 [0.32, 1.61]
Delayed	1.50 [0.59, 3.78]	1.18 [0.68, 2.05]	0.71 [0.22, 2.25]	1.08 [0.47, 2.49]	0.97 [0.38, 2.45]
Observations	1,495	1,495	1,495	1,495	1,495

Table Notes: *P<.05, **P<.01, ***P<.001. The model specifications were identical to the main effects regressions, except that the interaction effects between lifetime cannabis use and package features were added to each specification. Only past-year cannabis nonusers were included in this analysis. Only interaction effects are displayed; other coefficients are estimated but suppressed from the table. Standard errors were clustered at the state level.

© 2025 Cooper M et al. *JAMA Network Open.*

References

1. Cooper M, Shi Y. The impacts of packaging on preferences for cannabis edibles: A discrete choice experiment. *Int J Drug Policy*. Jun 2024;128:104453. doi:10.1016/j.drugpo.2024.104453
2. Cooper M, Shi Y. Appeal rating and visual attention associated with youth-appealing cannabis packaging: An eye-tracking experiment. *Drug Alcohol Depend*. Dec 1 2023;253:110992. doi:10.1016/j.drugalcdep.2023.110992
3. Shi Y, Pacula RL. Assessment of Recreational Cannabis Dispensaries' Compliance With Underage Access and Marketing Restrictions in California. *JAMA Pediatr*. Nov 1 2021;175(11):1178-1180. doi:10.1001/jamapediatrics.2021.2508
4. Blumenberg A. A cross-sectional assessment of package labels aimed at children of edible and leaf cannabis discarded in New York City. *Toxicol Commun*. 2022;6(1):78-84.
5. Ompad DC, Snyder KM, Sandh S, et al. Copycat and lookalike edible cannabis product packaging in the United States. *Drug Alcohol Depend*. Jun 1 2022;235:109409. doi:10.1016/j.drugalcdep.2022.109409
6. Tan ASL, Weinreich E, Padon A, et al. Presence of Content Appealing to Youth on Cannabis-Infused Edibles Packaging. *Subst Use Misuse*. 2022;57(8):1215-1219. doi:10.1080/10826084.2022.2069268
7. Barrus DG, Capogrossi KL, Cates SC, et al. Tasty THC: Promises and Challenges of Cannabis Edibles. *Methods Rep RTI Press*. Nov 2016;2016doi:10.3768/rtipress.2016.op.0035.1611
8. Hoeper S, Crosbie E, Holmes LM, et al. "The Perfect Formula:" Evaluating Health Claims, Products and Pricing on Cannabis Dispensary Websites in Two Recently Legalized States. *Subst Use Misuse*. 2022;57(8):1207-1214. doi:10.1080/10826084.2022.2069267
9. Schauer GL. Cannabis Policy in the United States: Implications for Public Health. *J Natl Cancer Inst Monogr*. Nov 28 2021;2021(58):39-52. doi:10.1093/jncimonographs/lgab016
10. Goodman S, Leos-Toro C, Hammond D. Do Mandatory Health Warning Labels on Consumer Products Increase Recall of the Health Risks of Cannabis? *Subst Use Misuse*. 2022;57(4):569-580. doi:10.1080/10826084.2021.2023186
11. Goodman S, Leos-Toro C, Hammond D. The impact of plain packaging and health warnings on consumer appeal of cannabis products. *Drug Alcohol Depend*. Dec 1 2019;205:107633. doi:10.1016/j.drugalcdep.2019.107633
12. Mutti-Packer S, Collyer B, Hodgins DC. Perceptions of plain packaging and health warning labels for cannabis among young adults: findings from an experimental study. *BMC Public Health*. Dec 10 2018;18(1):1361. doi:10.1186/s12889-018-6247-2
13. Kowitt SD, Yockey RA, Lee JGL, Jarman KL, Gourdet CK, Ranney LM. The Impact of Cannabis Packaging Characteristics on Perceptions and Intentions. *Am J Prev Med*. Nov 2022;63(5):751-759. doi:10.1016/j.amepre.2022.04.030
14. Byron MJ, Jeong M, Abrams DB, Brewer NT. Public misperception that very low nicotine cigarettes are less carcinogenic. *Tob Control*. Nov 2018;27(6):712-714. doi:10.1136/tobaccocontrol-2017-054124
15. Leos-Toro C, Fong GT, Hammond D. The efficacy of health warnings and package branding on perceptions of cannabis products among youth and young adults. *Drug Alcohol Rev*. May 2021;40(4):637-646. doi:10.1111/dar.13240

© 2025 Cooper M et al. *JAMA Network Open.*
