# Supplementary material for: Packaging of Cannabis Edibles, Health Warning Recall, and Perceptions Among Young Adults
Source: JAMA Netw Open. 2025 Apr 3;8(4):e253117. doi: 10.1001/jamanetworkopen.2025.3117 (PMC11969283; doi:10.1001/jamanetworkopen.2025.3117)
Supplement: Supplement 2. — Data Sharing Statement [file jamanetwopen-e253117-s002.pdf]

Data Sharing Statement

Cooper. Packaging of Cannabis Edibles, Health Warning Recall, and Perceptions Among Young Adults. *JAMA Netw Open*. Published April 03, 2025.
doi:10.1001/jamanetworkopen.2025.3117

Data
Data available: Yes
Data types: Deidentified participant data, Data dictionary
How to access data: Please contact Dr. Yuyan Shi (yus001@ucsd.edu) for data request.
When available: With publication

Supporting Documents
Document types: None

Additional Information
Who can access the data: Researchers whose proposed use of the data has been approved.
Types of analyses: Data will be made available for research purposes only.
Mechanisms of data availability: After approval of a research proposal and with a signed data access agreement.
